# An efficient and selective microwave-assisted Claisen-Schmidt reaction for the synthesis of functionalized benzalacetones

**DOI:** 10.1186/s40064-015-0985-8

**Published:** 2015-05-14

**Authors:** Anita Rayar, Maité Sylla-Iyarreta Veitía, Clotilde Ferroud

**Affiliations:** Laboratoire Chimie Moléculaire, génie des procédés chimiques et énergétiques (CMGPCE), EA 7341- Conservatoire National des Arts et Métiers, 2 rue Conté, 75003 Paris, France

**Keywords:** Green chemistry, Benzalacetones, Claisen-Schmidt reaction, Microwaves-assisted synthesis, Carbonyl compounds

## Abstract

A simple and direct method for the Claisen-Schmidt reaction to prepare functionalized α,β-unsaturated ketones has been developed. Microwave irradiation of aldehydes with acetone produces benzalacetones selectively without self-condensation product in very short reaction times and good yields.

**Electronic supplementary material:**

The online version of this article (doi:10.1186/s40064-015-0985-8) contains supplementary material, which is available to authorized users.

**Graphical Abstract:**
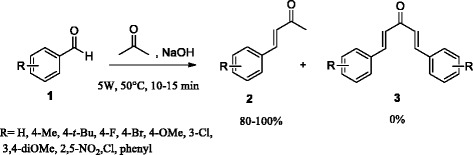
Graphics for use in the Table of Contents.

## Introduction

As part of our studies on the synthesis of anti-inflammatory compounds structurally related with coumarin skeleton, we have recently focused our attention on the synthesis of α,β-unsaturated ketones known as benzalacetones, which possess interesting properties for organic synthesis. Due to their conjugated system, benzalacetone and derivatives have been described as radical scavengers with potential antioxidant properties (Handayani & Arty 2008). Various methods of synthesis of this type of compounds have been described in the literature. The Claisen-Schmidt is one of the simplest condensation methods. The resulting β-hydroxycarbonyl compounds undergo a dehydration reaction, to afford the corresponding arylidene compounds. This reaction is typically catalyzed by acids (AlCl_3_ or HCl) and more often by base with or without solvent at room temperature or under conventional heating. However, in all these conditions, side reactions start decreasing the yield of the desired product and entail further purification steps (Drake & Allen 1923; Rahman et al. 2012; Jayapal et al. 2010).

In order to increase the yield and to avoid the formation of by-products, several protocols relative to a Claisen-Schmidt condensation have been reported using different catalysts: solid NaOH (Rahman et al. 2012), Ba(OH)_2_ (Aguilera et al. 1987), BF_3_.OEt_2_ (Narender & Papi Reddy 2007), NH_4_Cl (Pal 2013), BiCl_3_ (Kumar & Sandhu 2010), InCl_3_ (Deng & Ren 2003), TiCl_3_(SO_3_CF_3_) (Iranpoor et al. 1999), Yb(OTf)_3_ (Wang et al. 2004), FeCl_3_ (Zhang et al. 2003), molecular iodine (Sashidhara et al. 2009) or Co(II)-bipyridyl (Irie & Watanabe 1981). Sonochemical or microwaves irradiation methods have been reported using basic or acidic alumina (Esmaeili et al. 2005), Na_2_CO_3_-TBAB (PTC conditions) (Bogdal & Loupy 2008) and *bis*(p-methoxyphenyl)telluroxide (Zheng et al. 1997) as catalysts. These methods give good yields but suffer from long reaction times (e.g. 10 h with Cp_2_ZrH_2_/NiCl_2_) (Nakano et al. 1987), the use of expensive Lewis acids (e.g. amberlyst-15 and B_2_O_3_/ZrO_2_) (Pal et al. 2012), the use of hazardous catalyst or toxic solvents, variable reactions times and the formation of other byproducts (Gladkowski et al. 2013). Consequently, the development of an efficient and versatile method for the preparation of α,β-unsaturated ketones is of interest and there is a scope for further improvements towards mild reaction conditions and improved yields.

In pursuit of our investigations towards developing efficient methods for the synthesis of coumarin scaffolds, we were particularly interested in an efficient preparation of benzalacetones from acetone and aromatic substituted aldehydes. In this article, we report the microwave-assisted synthesis of functionalized benzalacetones in basic conditions. The advantages associated with the present protocol include the prevention of self-condensation product thus limiting the need for further purifications and ensuring short reaction times.

## Results and discussion

We decided to investigate a microwave-assisted synthesis of benzalacetones to increase reaction yields, to enhance the rate of reaction, thereby reducing time and decreasing by-products formation. Microwave activation for the synthesis of benzalacetones has not been widely described in the literature. Kappe et *al.* reported the aldol condensation of *p*-methoxybenzaldehyde with acetone using microwave activation but could not prevent self-condensation (Viviano et al. 2011).

Since a wide variety of aryl aldehydes are commercially available, Microwave activation would offer a higher degree of flexibility with regard to functional groups that can be introduced in the benzalacetone skeleton. For comparison, we’ll also present the results obtained using conventional heating conditions. The results are shown in Additional file 1: Table S1.

The conditions (temperature, NaOH and acetone equivalents) were optimized for the reaction of benzaldehyde as a model substrate with acetone used also as solvent. The conversion to the corresponding benzalacetone **2a** was optimal when by using 1.5 equiv of NaOH in acetone (13.6 equiv) at 40°C for 35 min. We consistently observed the presence of a byproduct, the dibenzalacetone **3** (ratio **2** vs **3**, 90:10, entry 2, Additional file 1: Table S1). We noticed that an excess of acetone (more than 10 equiv) was important to avoid the formation of a self-condensation product. The reactions carried out at room temperature were slightly slower than those performed at 40°C (generally 1 or 2 hours), and the results show no significant variation in the relative yields of **2a** versus **3a** (entries 1 and 2; Additional file 1: Table S1).

The Claisen-Schmidt conditions were successfully extended to the synthesis of several benzalacetones from various aldehydes. Reactions were monitored by GC-MS and conversions were determined by ^1^H NMR. The coupling constant between the olefinic protons in the ^1^H NMR spectrum (*J* = 16.2 Hz) confirmed the *E* configuration of the double bond C3-C4. The results obtained in conventional conditions are described in Additional file 1: Table S1. Despite the satisfactory yields obtained with conventional heating, the formation of dibenzalacetone **3** as byproduct was observed for all substrates (4-39%). It must be noted that benzalacetones **2** are unstable at room temperature and need to be store in cold conditions to further use. It should also be noted that longer reaction times result in the formation of other non-identified products.

Considering the results obtained under conventional heating and following our interest in establishing an efficient, rapid and selective access to benzalacetones, the Claisen-Schmidt condensations were undertaken under controlled microwave activation. In order to screen the microwave-assisted solvent-free conditions of benzalacetone synthesis, reactions were performed on the same aldehydes with 1.5 equiv of NaOH, in a Discover™ microwave synthesizer. The compounds were mixed in a sealed microwave reaction tube and irradiated for 10 to 30 minutes (5 W) with stirring at 50°C. When reactions were performed on a large scale, a flask equipped with a condenser was used. After irradiation, reactions were controlled by GC-MS analysis, and the purity of the desired products was evaluated by NMR spectroscopy.

Most of the microwave-assisted condensation reactions were performed at temperature of 40-50°C. In a preliminary study, we examined the temperature effect and we observed that condensation of the corresponding aldehydes with acetone occurred as soon as the temperature reached 50°C (in less than 5 minutes). In the case of 4-halogenated aldehydes (compounds **1d** and **1e**) reactions were carried out at 40°C to avoid the degradation of the reaction mixture. Decreasing the temperature caused no significant change in relative product yields and dibenzalacetone formation was never observed (entries 14 and 15, Additional file 1: Table S1).

The use of microwave activation resulted in a dramatic decrease of reaction times. The reactions were generally achieved within 10–15 min. The desired compounds were isolated with excellent yields (typically higher that 79% and often quantitative) and clean enough to be further used (based on ^1^H NMR analysis).

Additional file 1: Table S1 also shows the extension of these conditions to the synthesis of various benzalacetones. These microwave-assisted condensation reactions could be “directly scalable”. Identical yields were obtained on a 50 mg and 500 mg scale (compounds **2a**, **2b**, **2e** and **2f**, entries 10, 12, 16 and 18 respectively).

Taking into account these results we extended our study to other aldehydes (Additional file 2: Table S2). Good results were obtained with 3-chlorobenzaldehyde **1 g**. The reaction proceeded cleanly and the expected product **2 g** was selectively formed with a yield of 85%. However less satisfactory results were obtained with electron-withdrawing substituted aldehydes (compounds **1 h** and **1i**) for which dibenzalacetone formation was observed (Additional file 2: Table S2, entries 2 and 3). The lower yields obtained with electron-withdrawing substituted aldehydes, and particularly with 4-nitrobenzaldehyde (46%), can be attributed to product loss during work-up due to the emulsion formation. Additionally a further purification by chromatography was necessary to afford the desired compound **2 h** with a high purity confirmed by RMN analysis. The lower selectivities could be explained by the influence of nitro or triflouromethyl groups on reactivity of aldehydes **1 h** and **1i**. These electron-withdrawing groups increase the reactivity of the carbonyl function. The formed benzalacetone reacts faster with such activated aldehyde than with acetone leading to the formation of self-condensation product (compounds **3 h** and **3i**).

In the case of 3,4-dimethoxybenzaldehyde **1j**, the reaction proceeded in about 25 min and an irradiation of 50 W was required (entry 4, Additional file 2: Table S2). In the case of 5-chloro-2-nitro-aldehyde **1 k**, the reaction was easily performed at 5 W in 25 min with 90% yield. A quantitative reaction occured when naphthaldehyde **1 l** was used (entry 6, Additional file 2: Table S2) without any formation of self-condensation byproduct.

In conclusion, an efficient and selective general method has been developed for the synthesis of benzalacetones via a Claisen-Schmidt reaction using microwaves activation. In comparison with conventional heating methods, the desired compounds were obtained in shorter times and with excellent yields (in almost all cases, quantitative yields) and no further purification was required. Using microwaves activation, the formation of dibenzalacetone was never observed but in the case of electron-withdrawing substituted aldehydes. Further investigations concerning the use of functionalized benzalacetones as key intermediates in the synthesis of coumarin scaffolds assisted by microwave activation are currently underway.

### Experimental

Chemicals were used as received without special purification. All reactions were controlled by GC-MS analysis. Flash column chromatography was carried out when necessary using silica gel 60 (particle size 0.040-0.063 mm, Merck). ^1^H and ^13^C NMR spectra were acquired at room temperature on 400 MHz NMR spectrometer (100 MHz for ^13^C NMR). Chemical shifts are reported in δ units, parts per million (ppm). Coupling constants (J) are measured in hertz (Hz). Infrared spectra were recorded over the 400–4000 cm^−1^ range with an FTIR/ATR/ZnSe spectrometer. Microwaves experiments were performed using a single mode microwave reactor equipped with a 300 W power source and on-board infrared temperature sensor Additional file 3.

#### General procedure for the microwave-assisted syntheses

**Figure 1:**
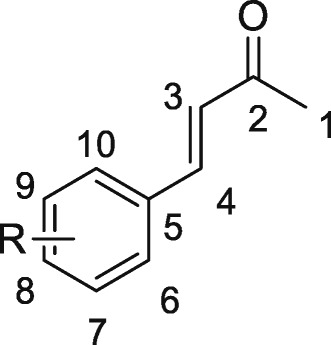
Convention adopted to assign signals of ^1^H and ^13^C-NMR spectra.

In a capped 10 mL MW-vessel, the aldehyde (50 mg, 1 equiv) and acetone (13.6 equiv) were mixed and then an aqueous solution of NaOH (0.6 g/cm^3^ of water) was added. The tube was positioned in the irradiation cavity and the mixture was stirred and heated at the temperature 40/50°C (measured with an IR temperature sensor), in the monomode microwave oven (5 W) for 10/15 min. After completion, upon cooling to ambient temperature, the conversion was directly controlled by GC-MS analysis. A simple consecutive work up (concentration of the reaction mixture and addition of 25 cm^3^ of water) affords the crude as an oil. The product was extracted with AcOEt (3×25 cm^3^). The organic layers were dried over anhydrous MgSO_4_, filtered and concentrated under reduced pressure to afford the corresponding benzalacetone. The purity of the final products was controlled by NMR. For the assignments of the NMR signals, we use the convention presented in Figure 1.

#### General procedure for conventional heating syntheses

To a mixture of appropriate aldehyde (50 mg, 1 equiv) and acetone (13.6 equiv) was added an aqueous solution of NaOH (0.6 g/cm^3^ of water). The mixture was stirred at 40°C and controlled by TLC and GC-MS. The same work up described above was used to afford the expected benzalacetones. When necessary the crude was purified by flash chromatography on silica gel. The purity of the final products was controlled by NMR. Additional file 4 and Additional file 5.

*(E)-4-phenylbut-3-en-2-one* (**2a**, C_10_H_10_O) *CAS : 1896-62-4* To a solution of benzaldehyde (1 equiv, 0.47 mmol), in acetone (13.6 equiv, 6.4 mmol) was added an aqueous solution of NaOH (28.2 mg/47 mm^3^ of water). The mixture was stirred under microwave irradiation. After work-up, the *(E)-*4-phenylbut-3-en-2-one was isolated as a yellow oil. Quantitative yield : 69 mg. R*f* = 0.40 SiO_2_ (CH_2_Cl_2_). *Spectroscopic data in accordance with literature reports* (McConville et al. 2009).

*(E)-4-p-tolylbut-3-en-2-one* (**2b**, C_11_H_12_O) *CAS : 4023-84-1*. To a solution of 4-methylbenzaldehyde (1 equiv, 0.42 mmol), in acetone (13.6 equiv, 5.8 mmol) was added an aqueous solution of NaOH (25.2 mg/42 mm^3^ of water). The mixture was stirred under microwave irradiation. After work-up, the *(E)-*4-*p*-tolylbut-3-en-2-one was isolated as a yellow oil. Yield : 64 mg (96%), R*f* = 0.31 SiO_2_ (CH_2_Cl_2_). *Spectroscopic data in accordance with literature reports* (McConville et al. 2009).

*(E)-4-(4-tert-butylphenyl)but-3-en-2-one* (**2c**, C_14_H_18_O) *CAS: 55047-62-6* To a solution of *4-tert*-butylbenzaldehyde (1 equiv, 0.31 mmol), in acetone (13.6 equiv, 4.2 mmol) was added an aqueous solution of NaOH (18.6 mg/31 mm^3^ of water). The mixture was stirred under microwave irradiation. After work-up, the *(E)-*4-(4-tert- butylphenyl)but-3-en-2-one was isolated as a yellow oil. Quantitative yield: 63 mg. R*f* = 0.33 SiO_2_ (cyclohexane/EtOAc : 9/1). ^1^H NMR (CDCl_3_, 400 MHz) *δ* (ppm) = 1.33 (s, 9H, H_10_), 2.37 (s, 3H, H_1_), 6.70 (d, 1H, J_3–4_ = 16.3 Hz, H_3_), 7.42 (d, 2H, J_ortho_ = 8.6 Hz, H_6_, H_6’_), 7.48 (d, 2H, J_ortho_ = 8.3 Hz, H_7_, H_7’_), 7.50 (d, 1H, J_4–3_ = 16.7 Hz, H_4_).^13^C NMR (CDCl_3_, 400 MHz) *δ* (ppm) = 27.4 (C_1_), 31.6 (C_10_,), 34.9 (C_9_), 125.9 (C_6_, C_6’_), 126.4 (C_3_), 128.1 (C_7_, C_7’_), 131.6 (C_5_), 143.5 (C_4_), 154.2 (C_8_), 198.6 (C = O). GC/MS : method 80; t_R_ = 10.28 min, *m/z*: [M]^+^ (202), 187 [M-CH_3_]^+^, 159 [M-COCH_3_]^+^. IR (ATR): = 3031 (ʋ_csp2-H_), 2961 (ʋ_csp3-H_), 1666 (ʋ _C=O_), 1604(ʋ _C=C_), 815 (δ_csp2-H, p-disubstitution_).

*(E)-4-(4-fluorophenyl)but-3-en-2-one* (**2d**, C_10_H_9_FO) *CAS : 65300-29-0* To a solution of 4-fluorobenzaldehyde (1 equiv, 0.40 mmol), in acetone (13.6 equiv, 5.4 mmol) was added an aqueous solution of NaOH 24 mg/40 mm^3^ of water). The mixture was stirred under microwave irradiation. After work-up, the *(E)-*4-(4-fluorophenyl)but-3-en-2-one was isolated as a yellow oil. Yield : 52.5 mg (80%) R*f* = 0.13 SiO_2_ (cyclohexane/EtOAc : 9/1). *Spectroscopic data in accordance with literature reports* (McConville et al. 2009).

*(E)-4-(4-bromophenyl)but-3-en-2-one* (**2e**, C_10_H_9_BrO) *CAS : 3815-31-4* To a solution of 4-bromobenzaldehyde (1 equiv, 0.27 mmol), in acetone (13.6 equiv, 3.7 mmol) was added an aqueous solution of NaOH 16.2 mg/27 mm^3^ of water). The mixture was stirred under microwave irradiation. After work-up, the *(E)-*4-(4-bromophenyl)but-3-en-2-one was isolated as a yellow oil. Yield: 48.3 mg (79%) R*f* = 0.22 SiO_2_ (cyclohexane/EtOAc : 9/1).^1^H NMR (CDCl_3_, 400 MHz) *δ* (ppm) = 2.38 (s, 3H, H_1_), 6.69 (d, 1H, J_3–4_ = 16.2 Hz, H_3_), 7.40 (d, 2H, J_ortho_ = 8.7 Hz, H_6_, H_6’_), 7.43 (d, 1H, J_4–3_ = 16.5 Hz, H_4_) ,7.54 (d, 2H, J_ortho_ = 8.7 Hz, H_7_, H_7’_). ^1^H *NMR data are in agreement with those previously reported* (Du et al. 2013).

*(E)-4-(4-methoxyphenyl)but-3-en-2-one* (**2f**, C_11_H_12_O_2_) *CAS : 3815-30-3* To a solution of 4-methoxybenzaldehyde (1 equiv, 0.37 mmol), in acetone (13.6 equiv, 5.0 mmol) was added an aqueous solution of NaOH (22.2 mg/37 mm^3^ of water). The mixture was stirred under microwave irradiation. After work-up, the *(E)-*4-(4-methoxyphenyl)but-3-en-2-one was isolated as a yellow oil. Quantitative Yield : 65 mg. R*f* = 0.53 SiO_2_ (cyclohexane/EtOAc : 9/1) *Spectroscopic data in accordance with literature reports* (McConville et al. 2009).

*(E)-4-(3-chlorophenyl)but-3-en-2-one***(2 g**, C_10_H_9_ClO**)***CAS 30626-02-9* To a solution of 3-chlorobenzaldehyde (1 equiv, 0.36 mmol), in acetone (13.6 equiv, 4.8 mmol) was added an aqueous solution of NaOH 21.3 mg/36 mm^3^ of water). The mixture was stirred under microwave irradiation. After work-up, the *(E)-*4-(3-chlorophenyl)but-3-en-2-one was isolated as a yellow oil. Yield : 56.1 mg (85%), R*f* = 0.49 SiO_2_ (cyclohexane/EtOAc : 8/2). *NMR data are in agreement with those previously reported* (He et al. 2013).

*(E)-4-(4-nitrophenyl)but-3-en-2-one* (**2 h**, C_10_H_9_NO_3_) *CAS : 30625-98-0* To a solution of 4-nitrobenzaldehyde (1 equiv, 0.33 mmol), in acetone (13.6 equiv, 4.5 mmol) was added an aqueous solution of NaOH 19.8 mg/33 mm^3^ of water). The mixture was stirred under microwave irradiation. After work-up, the residue was purified by chromatography column (cyclohexane/EtOAc : 7/3) and the *(E)-*4-(4-nitrophenyl)but-3-en-2-one was isolated as a yellow oil. Yield : 29 mg (46%), R*f* = 0.33 SiO_2_ (cyclohexane/EtOAc :9/1). ^1^H *NMR data are in agreement with those previously reported* (Du et al. 2013).

*(E)-4-(4-(trifluoromethyl)phenyl)but-3-en-2-one***(2i**, C_11_H_9_F_3_O**)***CAS : 115665-92-4* To a solution of 4-(trifluoromethyl)benzaldehyde (1 equiv, 0.29 mmol), in acetone (13.6 equiv, 3.9 mmol) was added an aqueous solution of NaOH 17.4 mg/29 mm^3^ of water). The mixture was stirred under microwave irradiation. After work-up, the *(E)-*4-(4-(trifluoromethyl)phenyl)but-3-en-2-one was isolated as a yellow oil. Yield : 47.8 mg (77%) R*f* = 0.49 SiO_2_ (cyclohexane/EtOAc : 9/1). ^1^H *NMR data are in agreement with those previously reported* (Du et al. 2013).

*(E)-4-(3,4-dimethoxyphenyl)but-3-en-2-one* (**2j**, C_12_H_14_O_3_) *CAS 60234-90-4* To a solution of 3,4-dimethoxybenzaldehyde (1 equiv, 0.30 mmol), in acetone (13.6 equiv, 4.1 mmol) was added an aqueous solution of NaOH 18 mg/30 mm^3^ of water). The mixture was stirred under microwave irradiation. After work-up, the *(E)-*4-(3,4-dimethoxyphenyl)but-3-en-2-one was isolated as a yellow oil. Yield : 61 mg (98%) R*f* = 0.19 SiO_2_ (cyclohexane/EtOAc : 8/2). *NMR data are in agreement with those previously reported* (He et al. 2013).

*(E)-4-(5-chloro-2-nitrophenyl)but-3-en-2-one* (**2 k**, C_10_H_8_ClNO_3_) *CAS 959058-78-7* To a solution of 5-chloro-2-nitrobenzaldehyde (1 equiv, 0.27 mmol), in acetone (13.6 equiv, 3.7 mmol) was added an aqueous solution of NaOH 16.2 mg/27 mm^3^ of water). The mixture was stirred under microwave irradiation. After work-up, *(E)-*4-(5-chloro-2-nitrophenyl)but-3-en-2-one was isolated as a yellow oil. Yield : 54.8 mg (90%) R*f* = 0.19 SiO_2_ (cyclohexane/EtOAc : 8/2). *NMR data are in agreement with those previously reported* (Okuro et al. 2011).

*(E)-4-(naphthalen-1-yl)but-3-en-2-one* (**2 l**, C_14_H_12_O) To a solution of 1-naphtaldehyde (1 equiv, 0.32 mmol), in acetone (13.6 equiv, 4.4 mmol) was added an aqueous solution of NaOH 19.2 mg/32 mm^3^ of water). The mixture was stirred under microwave irradiation. After work-up, the *(E)-*4-(naphthalen-1-yl)but-3-en-2-one was isolated as a yellow oil. Quantitative yield : 63 mg. R*f* = 0.55 SiO_2_ (cyclohexane/EtOAc : 8/2). ^1^H *NMR data are in agreement with those previously reported* (Du et al. 2013).

## Additional files

Additional file 1: Table S1. Preparation of benzalacetones using conventional heating and microwaves conditions^a^. Additional file 2: Table S2. Preparation of benzalacetones using microwaves conditions^a^. Additional file 3:
**Full materials and methods.**
Additional file 4:
**Full spectral data of synthesized compounds.**
Additional file 5:
**Proton NMR spectra of compounds 2a-2l.**
