# Supplementary material for: An efficient and selective microwave-assisted Claisen-Schmidt reaction for the synthesis of functionalized benzalacetones
Source: Springerplus. 2015 May 14;4:221. doi: 10.1186/s40064-015-0985-8 (PMC4456587; doi:10.1186/s40064-015-0985-8)
Supplement: Additional file 1: Table S1. — Preparation of benzalacetones using conventional heating and microwaves conditionsa. [file 40064_2015_985_MOESM1_ESM.docx]

**Additional file 1**

**Table 1**. Preparation of benzalacetones using conventional heating and microwaves conditions ^a^

| Entry | (aldehyde **1**) R | Conventional heating  (Temp/°C, Time/h) | Ratio (%)^b^  **2** **3** | | Product^c^ (Yield %) | Entry | | | MW Conditions  (Power,Temp/°C, Time/min) | | Ratio (%)^b^  **2** **3** | | Product^c^ (Yield %) | |
| --- | --- | --- | --- | --- | --- | --- | --- | --- | --- | --- | --- | --- | --- | --- |
| 1 | **(1a)** 4-H | 20 °C, 1 h | 92 | 8 | **2a** (85) | | 9 | 5 W, 50 °C, 10 min | | 100 | | 0 | | **2a (**100) |
| 2 |  | 40 °C, 0.35 h | 90 | 10 | **2a** (82) | | 10 | 5 W, 50 °C, 15 min^d^ | | 100 | | 0 | | **2a (**100) |
| 3 | **(1b)** 4-Me | 20°C, 2 h | 93 | 7 | **2b** (73) | | 11 | 5 W, 50 °C, 10 min | | 100 | | 0 | | **2b (**96) |
| 4 |  | 40°C, 1.5 h | 95 | 5 | **2b** (77) | | 12 | 5 W, 50 °C, 15 min^d^ | | 100 | | 0 | | **2b (**98) |
| 5 | **(1c)** 4-*t*-Bu | 40°C, 1.1 h | 85 | 5 | **2c** (70) | | 13 | 5 W, 50 °C, 15 min | | 100 | | 0 | | **2c (**100) |
| 6 | **(1d)** 4-F | 40 °C, 1.5 h | 82 | 18 | **2d** (42) | | 14 | 5 W, 40°C, 10 min | | 100 | | 0 | | **2d** (80) |
| 7 | **(1e)** 4-Br | 40 °C, 2 h | 61 | 39 | **2e** (12) | | 15 | 5 W, 40°C, 10 min | | 100 | | 0 | | **2e** (79) |
|  |  |  |  |  |  | | 16 | 5 W, 40 °C, 15 min^d^ | | 100 | | 0 | | **2e** (85) |
| 8 | **(1f)** 4-OMe | 40 °C, 1.5 h | 96 | 4 | **2f** (43) | | 17 | 5 W, 50°C, 15 min | | 100 | | 0 | | **2f** (100) |
|  |  |  |  |  |  | | 18 | 5 W, 50 °C, 35 min^d^ | | 100 | | 0 | | **2f** (100) |

^a^ Reactions performed at scale of 50 mg , ^b^ Ratio was determined by ^1^H NMR, ^c^ Isolated yield after work-up. The purity was controlled by ^1^H NMR

^d^ Reactions performed at scale of 500 mg
