# Supplementary material for: An efficient and selective microwave-assisted Claisen-Schmidt reaction for the synthesis of functionalized benzalacetones
Source: Springerplus. 2015 May 14;4:221. doi: 10.1186/s40064-015-0985-8 (PMC4456587; doi:10.1186/s40064-015-0985-8)
Supplement: Additional file 2: Table S2. — Preparation of benzalacetones using microwaves conditionsa. [file 40064_2015_985_MOESM2_ESM.docx]

**Additional file 2**

**Table 2** Preparation of benzalacetones using microwaves conditions ^a^

| Entry | (aldehyde **1**) R | MW Conditions  (Power/W,Temp/°C, Time/min) | Ratio (%)^b^  **2** **3** | | Product ^c^  (Yield %) | |
| --- | --- | --- | --- | --- | --- | --- |
| 1 | **(1g)** 3-Cl | 5 W, 50 °C, 10 min | 100 | 0 | | **2g** (85) |
| 2 | **(1h)** 4-NO_2_ | 10 W, 50°C, 10 min | 70 | 30 | | **2h** (46) |
| 3 | **(1i)** 4-CF_3_ | 5 W, 50°C, 10 min | 80 | 20 | | **2i** (77) |
| 4 | **(1j)** 3,4-di-OMe | 50 W, 50 °C, 25 min | 100 | 0 | | **2j** (98) |
| 5 | **(1k)** 2-NO_2_, 5-Cl | 5W, 50 °C, 25 min | 88^d^ | 0 | | **2k** (90) |
| 6 | **(1l)** phenyl | 5W, 50 °C, 20 min | 100 | 0 | | **2l** (100) |

^a^ Reactions performed at scale of 50 mg ,

^b^ Ratio was determined by ^1^H NMR,

^c^ Isolated yield after work-up. The purity was controlled by ^1^H NMR,

^d^ 12% of non-reacted aldehyde recovered
