# Supplementary material for: An efficient and selective microwave-assisted Claisen-Schmidt reaction for the synthesis of functionalized benzalacetones
Source: Springerplus. 2015 May 14;4:221. doi: 10.1186/s40064-015-0985-8 (PMC4456587; doi:10.1186/s40064-015-0985-8)
Supplement: Additional file 3: — Full materials and methods. [file 40064_2015_985_MOESM3_ESM.docx]

**Additional file 3**

**Materials and Methods**

All reagents were obtained from commercial sources unless otherwise noted, and used as received. Conventional heated experiments were conducted using thermostatically controlled oil baths and were performed in oven-dried glassware. All reactions were controlled by analytical thin layer chromatography (TLC) and GC-MS analysis. TLC were performed on aluminium sheets precoated silica gel plates (60 F_254_, Merck). TLC plates were visualized using irradiation with light at 254 nm or in an iodine chamber as appropriate. Flash column chromatography was carried out when necessary using silica gel 60 (particle size 0.040-0.063 mm, Merck).

^1^H and ^13^C-NMR spectra were acquired on a Bruker BioSpin GmbH spectrometer 400 MHz, at room temperature. Chemical shifts are reported in δ units, parts per million (ppm). Coupling constants (J) are measured in hertz (Hz). Splitting patterns are designed as follows: s, singlet; d, doublet; m, multiplet. Various 2D techniques and DEPT experiments were used to establish the structures and to assign the signals. GC-MS analysis were performed with an Agilent 6890N instrument equipped with a dimethyl polysiloxane capillary column (12 m x 0.20 mm) and an Agilent 5973N MS detector-column temperature *gradient* 80-300°C (method 80): 80°C (1 min); 80°C to 300°C (12.05°C/min); 300°C (2 min); Infrared spectra were recorded over the 400-4000 cm^-1^ range with an Agilent Technologies Cary 630 FTIR/ ATR/ ZnSe spectrometer.
