# Supplementary material for: An efficient and selective microwave-assisted Claisen-Schmidt reaction for the synthesis of functionalized benzalacetones
Source: Springerplus. 2015 May 14;4:221. doi: 10.1186/s40064-015-0985-8 (PMC4456587; doi:10.1186/s40064-015-0985-8)
Supplement: Additional file 4: — Full spectral data of synthesized compounds. [file 40064_2015_985_MOESM4_ESM.docx]

**Additional file 4**

Full spectral data of synthesized compounds

*(E)-4-phenylbut-3-en-2-one* (**2a**, C_10_H_10_O) *CAS : 1896-62-4* To a solution of benzaldehyde (1 equiv, 0.47 mmol), in acetone (13.6 equiv, 6.4 mmol) was added an aqueous solution of NaOH (28.2 mg / 47 mm^3^ of water). The mixture was stirred under microwaves irradiation. After work-up, the *(E)-*4-phenylbut-3-en-2-one was isolated as a yellow oil. Yield : 69 mg, Qtf. R*f* = 0.40 SiO_2_ (CH_2_Cl_2_).^1^H NMR (CDCl_3_, 400 MHz) *δ* (ppm) = 2.38 (s, 3H, H_1_), 6.72 (d, 1H, J_2-3_ = 16.28 Hz, H_3_), 7.38-7.42 (m, 3H, H_7_, H_7’_, H_8_), 7.51 (d, 1H, J_3-4_ = 16.4 Hz, H_4_), 7.53-7.56 (m, 2H, H_6_, H_6’_).^13^C NMR (CDCl_3_, 400 MHz) *δ* (ppm) = 27.6 (C_1_), 127.2 (C_3_), 128.3 (C_6_, C_6’_), 129.0 (C_7_, C_7’_), 130.6 (C_8_), 134.5 (C_5_), 143.5 (C_4_), 198.5 (C=O). GC/MS : method 80; t_R_ = 6.09 min, *m/z*: [M]^+^ (146), 131 [M-CH_3_]^+^, 103 [M-COCH_3_]^+^. IR (ATR): = 3061, 3029 (ʋ_csp2-H_), 1666 (ʋ _C=O_), 1607 (ʋ _C=C_).

*(E)-4-p-tolylbut-3-en-2-one* (**2b**, C_11_H_12_O) *CAS : 4023-84-1*. To a solution of 4-methylbenzaldehyde (1 equiv, 0.42 mmol), in acetone (13.6 equiv, 5.8 mmol) was added an aqueous solution of NaOH (25.2 mg / 42 mm^3^ of water). The mixture was stirred under microwaves irradiation. After work-up, the *(E)-*4-*p*-tolylbut-3-en-2-one was isolated as a yellow oil. Yield : 64 mg (96%), R*f* = 0.31 SiO_2_ (CH_2_Cl_2_).^1^H NMR (CDCl_3_, 400 MHz) *δ* (ppm) = 2.36 (s, 3H, H_1_), 2.37 (s, 3H, H_9_), 6.67 (d, 1H, J_3-4_=16.3 Hz, H_3_), 7.19 (d, 2H, J_ortho_=7.9 Hz, H_6_, H_6’_), 7.44 (d, 2H, J_ortho_= 8.3 Hz, H_7_, H_7’_), 7.47 (d, 1H, J_3-4_ = 16.3 Hz, H_4_).^13^C NMR (CDCl_3_, 400 MHz) *δ* (ppm) = 21.5 (C_1_), 27.5 (C_9_), 126.3 (C_3_), 128.3 (C_7_, C_7’_ ), 129.7 (C_6_, C_6’_), 131.7 (C_5_), 141.0 (C_8_), 143.5 (C_4_), 198.5 (C=O). GC/MS : method 80; t_R_ = 7.53 min, *m/z*: [M]^+^ (160), 145 [M-CH_3_]^+^, 117 [M-COCH_3_]^+^. IR (ATR): = 3025 (ʋ_csp2-H_), 2922 (ʋ_csp3-H_), 1663 (ʋ _C=O_), 1608(ʋ _C=C_), 798 (δ_csp2-H, p-disubstitution_).

*(E)-4-(4-tert-butylphenyl)but-3-en-2-one* (**2c**, C_14_H_18_O) *CAS : 55047-62-6* To a solution of *4-tert*-butylbenzaldehyde (1 equiv, 0.31 mmol), in acetone (13.6 equiv, 4.2 mmol) was added an aqueous solution of NaOH (18.6 mg / 31 mm^3^ of water). The mixture was stirred under microwaves irradiation. After work-up, the *(E)-*4-(4-tert- butylphenyl)but-3-en-2-one was isolated as a yellow oil. Yield : 63 mg, Qtf. R*f* = 0.33 SiO_2_ (cyclohexane/EtOAc : 9/1). ^1^H NMR (CDCl_3_, 400 MHz) *δ* (ppm) = 1.33 (s, 9H, H_10_), 2.37 (s, 3H, H_1_), 6.70 (d, 1H, J_3-4_= 16.3 Hz, H_3_), 7.42 (d, 2H, J_ortho_= 8.6 Hz, H_6_, H_6’_), 7.48 (d, 2H, J_ortho_= 8.3 Hz, H_7_, H_7’_), 7.50 (d, 1H, J_4-3_ = 16.7 Hz, H_4_).^13^C NMR (CDCl_3_, 400 MHz) *δ* (ppm) = 27.4 (C_1_), 31.6 (C_10_,), 34.9 (C_9_), 125.9 (C_6_, C_6’_), 126.4 (C_3_), 128.1 (C_7_, C_7’_), 131.6 (C_5_), 143.5 (C_4_), 154.2 (C_8_), 198.6 (C=O). GC/MS : method 80; t_R_ = 10.28 min, *m/z*: [M]^+^ (202), 187 [M-CH_3_]^+^, 159 [M-COCH_3_]^+^. IR (ATR): = 3031 (ʋ_csp2-H_), 2961 (ʋ_csp3-H_), 1666 (ʋ _C=O_), 1604(ʋ _C=C_), 815 (δ_csp2-H, p-disubstitution_).

*(E)-4-(4-fluorophenyl)but-3-en-2-one* (**2d**, C_10_H_9_FO) *CAS : 65300-29-0* To a solution of 4-fluorobenzaldehyde (1 equiv, 0.40 mmol), in acetone (13.6 equiv, 5.4 mmol) was added an aqueous solution of NaOH 24 mg / 40 mm^3^ of water). The mixture was stirred under microwaves irradiation. After work-up, the *(E)-*4-(4-fluorophenyl)but-3-en-2-one was isolated as a yellow oil. Yield : 52.5 mg (80%) R*f* = 0.13 SiO_2_ (cyclohexane/EtOAc :9/1).^1^H NMR (CDCl_3_, 400 MHz) *δ* (ppm) = 2.35 (s, 3H, H_1_), 6.62 (d, 1H, J_3-4_= 16.2 Hz, H_3_), 7.07 (dd, 2H, J_ortho_= J_H-F_ = 8.6 Hz, H_7_, H_7’_), 7.45 (d, 1H, J_4-3_ = 16.3 Hz, H_4_) ,7.51 (ddd, 2H, J_ortho_= 8.9 Hz, J_H-F_ = 5.4 Hz, J_para_= 0.48 Hz).^13^C NMR (CDCl_3_, 400 MHz) *δ* (ppm) = 27.6 (C_1_), 116.1 (d, 2C, J_C-F_ = 21.8 Hz, C_7_, C_7’_), 116.2 (C_3_), 130.1 (d, 2C, J_C-F_ = 8.5 Hz, C_6_, C_6’_), 130.6 (d, 1H, J_C-F_ = 3.2 Hz, C_5_), 142.09 (C_4_), 164.0 (d, 1H, J_C-F_ = 250.4 Hz, C_9_), 198.2 (C=O). GC/MS : method 80; t_R_ = 6.23 min, *m/z*: [M]^+^ (164), 149 [M-CH_3_]^+^, 121 [M-COCH_3_]^+^. IR (ATR): = 3025 (ʋ_csp2-H_), 2926 (ʋ_csp3-H_), 1666 (ʋ _C=O_),1597, 1507 (ʋ _C=C_), 1225 (ʋ _C-F_ ), 815 (δ_csp2-H, p-disubstitution_).

*(E)-4-(4-bromophenyl)but-3-en-2-one* (**2e**, C_10_H_9_BrO) *CAS : 3815-31-4* To a solution of 4-bromobenzaldehyde (1 equiv, 0.27 mmol), in acetone (13.6 equiv, 3.7 mmol) was added an aqueous solution of NaOH 16.2 mg / 27 mm^3^ of water). The mixture was stirred under microwaves irradiation. After work-up, the *(E)-*4-(4-bromophenyl)but-3-en-2-one was isolated as a yellow oil. Yield: 48.3 mg (79%) R*f* = 0.22 SiO_2_ (cyclohexane/EtOAc :9/1).^1^H NMR (CDCl_3_, 400 MHz) *δ* (ppm) = 2.38 (s, 3H, H_1_), 6.69 (d, 1H, J_3-4_= 16.2 Hz, H_3_), 7.40 (d, 2H, J_ortho_= 8.7 Hz, H_6_, H_6’_), 7.43 (d, 1H, J_4-3_ = 16.5 Hz, H_4_) ,7.54 (d, 2H, J_ortho_= 8.7 Hz, H_7_, H_7’_). ^13^C NMR (CDCl_3_, 400 MHz) *δ* (ppm) = 27.7 (C_1_), 124.8 (C_5_), 127.5 (C_3_), 129.6 (C_6_, C_6’_), 131.8 (C_5_), 132.2 (C_7_, C_7’_), 141.9 (C_4_), 196.6 (C=O). GC/MS : method 80; t_R_ = 9.41 min, *m/z*: [M]^+^ (226), 165 [M-CH_3_]^+^, 183 [M-COCH_3_]^+^. IR (ATR): = 3050 (ʋ_csp2-H_), 2920 (ʋ_csp3-H_), 1655 (ʋ _C=O_),1607, 1585, 1486, (ʋ _C=C_),804 (δ_csp2-H, p-disubstitution_).

*(E)-4-(4-methoxyphenyl)but-3-en-2-one* (**2f**, C_11_H_12_O_2_) *CAS : 3815-30-3* To a solution of 4-methoxybenzaldehyde (1 equiv, 0.37 mmol), in acetone (13.6 equiv, 5.0 mmol) was added an aqueous solution of NaOH (22.2 mg / 37 mm^3^ of water). The mixture was stirred under microwaves irradiation. After work-up, the *(E)-*4-(4-methoxyphenyl)but-3-en-2-one was isolated as a yellow oil. Yield : 65 mg, Qtf. R*f* = 0.53 SiO_2_ (cyclohexane/EtOAc :9/1) ^1^H NMR (CDCl_3_, 400 MHz) *δ* (ppm) = 2.33 (s, 3H, H_1_), 3.81 (s, 3H, H_9_), 6.58 (d, 1H, J_3-4_=16.2 Hz, H_2_), 6.88 (d, 2H, J_ortho_=8.8 Hz, H_7_, H_7’_), 7.45 (d, 1H, J_4-3_ = 16.6 Hz, H_4_) , 7.47 (d, 2H, J_ortho_ = 8.9 Hz, H_6_, H_6’_).^13^C NMR (CDCl_3_, 400 MHz) *δ* (ppm) = 27.4 (C_1_), 55.4 (C_9_), 114.4 (C_7_, C_7’_), 125.0 (C_3_), 127.0 (C_5_), 129.9 (C_6_, C_6’_), 143.5 (C_4_), 161.6 (C_8_), 198.5 (C=O). GC/MS : method 80; t_R_ = 9.49 min, *m/z*: [M]^+^ (176), 161 [M-CH_3_]^+^, 133 [M-COCH_3_]^+^. IR (ATR): = 3005 (ʋ_csp2-H_), 2919 (ʋ_csp3-H_), 2840 (ʋ _O-Me_), 1680 (ʋ _C=O_), 1662 (ʋ _C=C_), 1588, 1575, 1509,1464 (ʋ_csp2ar-C_), 816 (δ_csp2-H, p-disubstitution_).

*(E)-4-(3-chlorophenyl)but-3-en-2-one* **(2g**, C_10_H_9_ClO**)** *CAS 30626-02-9* To a solution of 3-chlorobenzaldehyde (1 equiv, 0.36 mmol), in acetone (13.6 equiv, 4.8 mmol) was added an aqueous solution of NaOH 21.3 mg / 36 mm^3^ of water). The mixture was stirred under microwaves irradiation. After work-up, the *(E)-*4-(3-chlorophenyl)but-3-en-2-one was isolated as a yellow oil. Yield : 56.1 mg (85%), R*f* = 0.49 SiO_2_(cyclohexane/EtOAc :8/2). ^1^H NMR (CDCl_3_, 400 MHz) *δ* (ppm) = 2.38 (s, 3H, H_1_), 6.70 (d, 1H, J_3-4_= 16.3 Hz, H_3_), 7.34-7.38 (m, 2H, H_7_, H_8_), 7.40 (m, 1H, H_6_) ,7.43 (d, 1H, J_4-3_= 16.2 Hz, H_4_), 7.52 (m, 1H, H_10_). *NMR data are in agreement with those previously reported* [22]. GC/MS : method 80; t_R_ = 5.89 min, *m/z*: [M]^+^ (180), 165 [M-CH_3_]^+^, 137 [M-COCH_3_]^+^. IR (ATR): = 3063 (ʋ_csp2-H_), 2923 (ʋ_csp3-H_), 1667 (ʋ _C=O_),1594, 1568,1475 (ʋ _C=C_), 780 (δ_csp2-H, m-disubstitution_).

*(E)-4-(4-nitrophenyl)but-3-en-2-one* (**2h**, C_10_H_9_NO_3_) *CAS : 30625-98-0* To a solution of 4-nitrobenzaldehyde (1 equiv, 0.33 mmol), in acetone (13.6 equiv, 4.5 mmol) was added an aqueous solution of NaOH 19.8 mg / 33 mm^3^ of water). The mixture was stirred under microwaves irradiation. After work-up, the residue was purified by chromatography column (cyclohexane/EtOAc :7/3) and the *(E)-*4-(4-nitrophenyl)but-3-en-2-one was isolated as a yellow oil. Yield : 29 mg (46%), R*f* = 0.33 SiO_2_ (cyclohexane/EtOAc :9/1).^1^H NMR (CDCl_3_, 400 MHz) *δ* (ppm) = 2.43 (s, 3H, H_1_), 6.82 (d, 1H, J_3-4_= 16.3 Hz, H_3_), 7.53 (d, 1H, J_4-3_= 16.3 Hz, H_4_), 7.7 (d, 2H, J_6-6’_ = 8.9 Hz, H_6_, H_6’_), 8.26 (d, 2H, J_7-7’_= 8.7 Hz, H_7_, H_7_’). GC/MS: method 80; t_R_ = 6.90 min, *m/z*: [M]^+^ (191), 176 [M-CH_3_]^+^. IR (ATR): = 3084 (ʋ_csp2-H_), 2923 (ʋ_csp3-H_), 1591, (ʋ _Car=Car_), 1512 (ʋ _asNO2_), 1342 (ʋ _sNO2_).

*(E)-4-(4-(trifluoromethyl)phenyl)but-3-en-2-one* **(2i**, C_11_H_9_F_3_O**)** *CAS :115665-92-4* To a solution of 4-(trifluoromethyl)benzaldehyde (1 equiv, 0.29 mmol), in acetone (13.6 equiv, 3.9 mmol) was added an aqueous solution of NaOH 17.4 mg / 29 mm^3^ of water). The mixture was stirred under microwaves irradiation. After work-up, the *(E)-*4-(4-(trifluoromethyl)phenyl)but-3-en-2-one was isolated as a yellow oil. Yield : 47.8 mg (77%) R*f* = 0.49 SiO_2_ (cyclohexane/EtOAc :9/1).^1^H NMR (CDCl_3_, 400 MHz) *δ* (ppm) = 2.39 (s, 3H, H_1_), 6.76 (d, 1H, J_3-4_= 16.3 Hz, H_3_), 7.47 (d, 2H, J_6-6’_= 8.0 Hz, H_6_, H_6’_), 7.51 (d, 1H, J_4-3_ = 16.3 Hz, H_4_) ,7.59 (d, 2H, J_7-7’_= 8.0 Hz, H_7_,H_7’_), 7.46 (s,CF_3_). ^1^H *NMR data are in agreement with those previously reported* [23].GC/MS : method 80; t_R_ = 4.54 min, *m/z*: [M]^+^ (214), 199 [M-CH_3_]^+^, 171 [M-COCH_3_]^+^. IR (ATR): =3051 (ʋ_csp2-H_), 2976 (ʋ_csp3-H_), 1321 (ʋ _C-F_).

*(E)-4-(3,4-dimethoxyphenyl)but-3-en-2-one* (**2j**, C_12_H_14_O_3_) *CAS 60234-90-4* To a solution of 3,4-dimethoxybenzaldehyde (1 equiv, 0.30 mmol), in acetone (13.6 equiv, 4.1 mmol) was added an aqueous solution of NaOH 18 mg / 30 mm^3^ of water). The mixture was stirred under microwaves irradiation. After work-up, the *(E)-*4-(3,4-dimethoxyphenyl)but-3-en-2-one was isolated as a yellow oil. Yield : 61 mg (98%) R*f* = 0.19 SiO_2_(cyclohexane/EtOAc : 8/2) ^1^H NMR (CDCl_3_, 400 MHz) *δ* (ppm) = 2.56 (s, 3H, H_1_), 4.11 (s, 6H, H_8_, H_9_), 6.79 (d, 1H, J_3-4_= 16.2 Hz, H_3_), 7.06 (d, 1H, J_7-6_= 8.3 Hz, H_7_, H_6_) ,7.26 (m, 1H, H_10_), 7.31 (d,1H, J_6-7_= 8.3 Hz, H_6_, H_7_), 7.65 (d, 1H, J_4-3_= 16.2 Hz, H_4_). *NMR data are in agreement with those previously reported* [22]. GC/MS : method 80; t_R_ = 8.01 min, *m/z*: [M]^+^ (206), 191 [M-CH_3_]^+^, 163 [M-COCH_3_]^+^. IR (ATR): = 3012 (ʋ_csp2-H_), 2945-2919 (ʋ_csp3-H_), 2841 (ʋ OMe), 1666 (ʋ _C=O_), 1594, 1510,1453 (ʋ _Car=Car_), 802 (δ_csp2-H_).

*(E)-4-(5-chloro-2-nitrophenyl)but-3-en-2-one* (**2k**, C_10_H_8_ClNO_3_) *CAS 959058-78-7* To a solution of 5-chloro-2-nitrobenzaldehyde (1 equiv, 0.27 mmol), in acetone (13.6 equiv, 3.7 mmol) was added an aqueous solution of NaOH 16.2 mg / 27 mm^3^ of water). The mixture was stirred under microwaves irradiation. After work-up, *(E)-*4-(5-chloro-2-nitrophenyl)but-3-en-2-one was isolated as a yellow oil. Yield : 54.8 mg (90%) R*f* = 0.19 SiO_2_(cyclohexane/EtOAc : 8/2). ^1^H NMR (CDCl_3_, 400 MHz) *δ* (ppm) = 2.36 (s, 3H, H_1_), 6.49 (d, 1H, J_3-4_= 16.2 Hz, H_3_), 7.25 (m, 2H, J= 8.8 Hz, H_6_, H_8_),7.88 (d, 1H, J_4-3_= 16.2 Hz, H_4_), 8.00 (d,1H, J= 8.8 Hz, H_9_). *NMR data are in agreement with those previously reported* [24]. GC/MS : method 80; t_R_ = 7.80 min, *m/z*: 182 [M-COCH_3_]^+^. IR (ATR): = 3074 (ʋ_csp2-H_), 2925 (ʋ_csp3-H_), 1715 (ʋ _C=O_), 1600, 1563, 1521 (ʋ _Car=Car_), 902, 822 (δ_csp2-H_).

*(E)-4-(naphthalen-1-yl)but-3-en-2-one* (**2l**, C_14_H_12_O) To a solution of 1-naphtaldehyde (1 equiv, 0.32 mmol), in acetone (13.6 equiv, 4.4 mmol) was added an aqueous solution of NaOH 19.2 mg / 32 mm^3^ of water). The mixture was stirred under microwaves irradiation. After work-up, the *(E)-*4-(naphthalen-1-yl)but-3-en-2-one was isolated as a yellow oil. Yield : 63 mg, Qtf. R*f* = 0.55 SiO_2_ (cyclohexane/EtOAc : 8/2) ^1^H NMR (CDCl_3_, 400 MHz) *δ* (ppm) = 2.46 (s, 3H, H_1_), 6.81 (d, 1H, J_3-4_= 15.9 Hz, H_3_), 7.26-7.61 (m, 3H, H_12_, H_13_, H_14_) ,7.77 (d, 1H, J= 7.1 Hz, H_10_), 7.89 (d, 2H, J= 8.3 Hz, H_8_,H_9_), 8.17 (d, 1H, J= 8.4 Hz, H_7_), 8.37 (d, 1H, J_4-3_= 15.9 Hz, H_4_). ^1^H *NMR data are in agreement with those previously reported* [23]. GC/MS: method 80; t_R_ = 8.05 min, *m/z*: [M]^+^ (196), 181 [M-CH_3_]^+^, 153 [M-COCH_3_]^+^. IR (ATR): = 3049 (ʋ_csp2-H_), 2971 (ʋ_csp3-H_), 1668 (ʋ _C=O_), 1599, 1510 (ʋ _Car=Car_), 778, 729 (δ_csp2-H_).
