# Supplementary material for: An efficient and selective microwave-assisted Claisen-Schmidt reaction for the synthesis of functionalized benzalacetones
Source: Springerplus. 2015 May 14;4:221. doi: 10.1186/s40064-015-0985-8 (PMC4456587; doi:10.1186/s40064-015-0985-8)

**Additional file 5**

Proton NMR spectra of compounds **2a**-**2l**.

***(E)-*4-phenylbut-3-en-2-one (2a)**

***
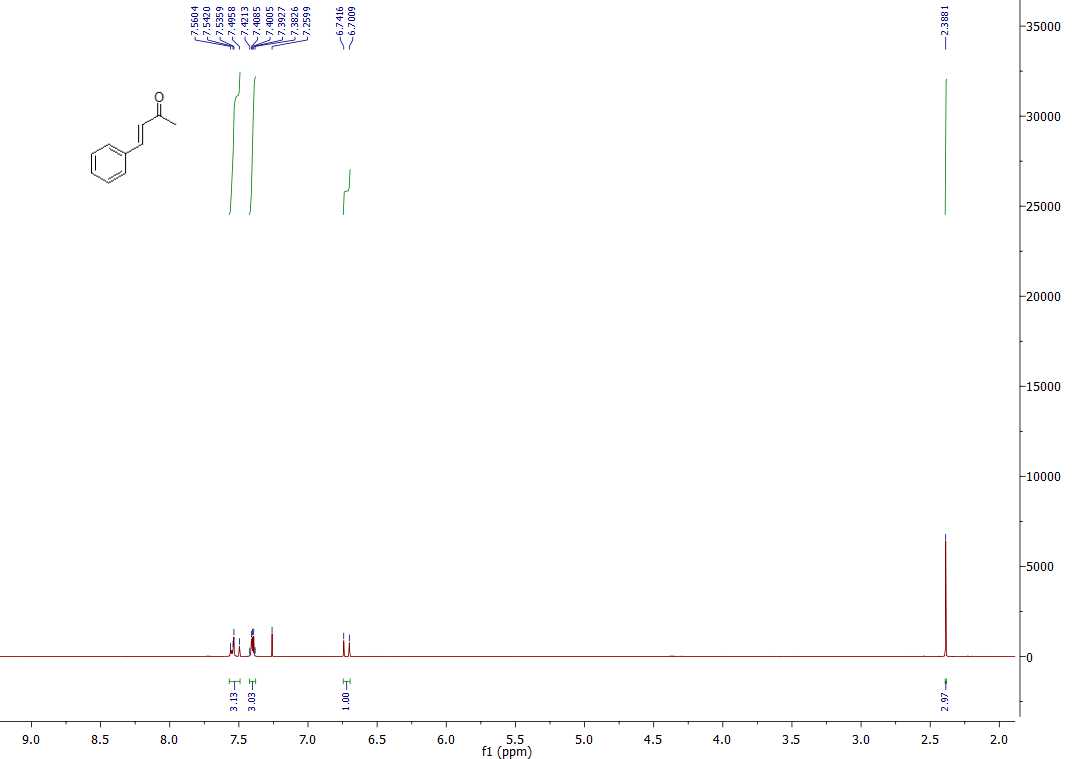
***

***(E)-*4-*p*-tolylbut-3-en-2-one (2b)**

***
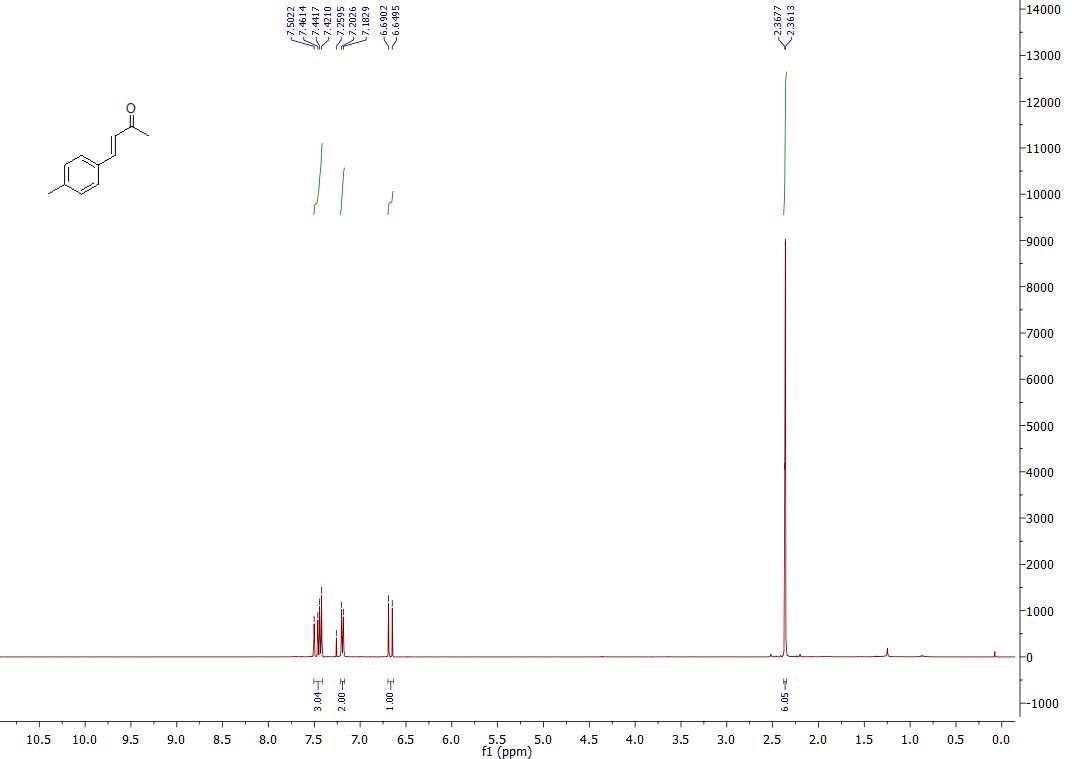
***

***(E)-*4-(4-tert-butylphenyl)but-3-en-2-one (2c)**

***
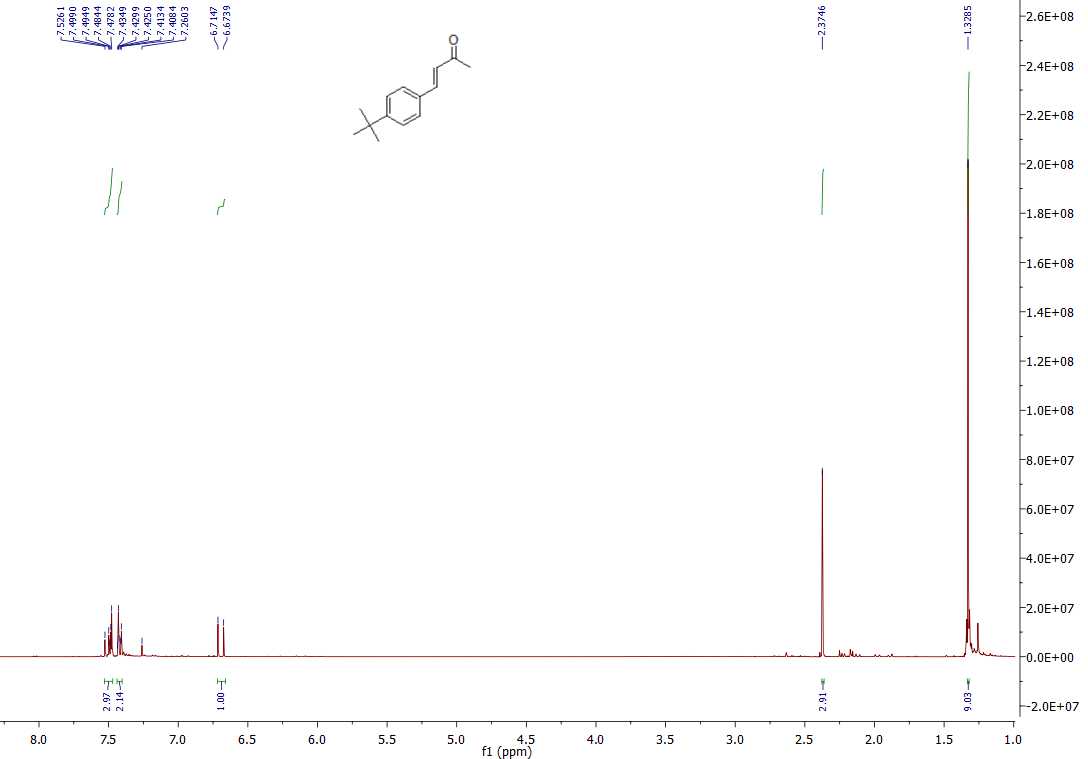
***

***(E)-*4-(4-fluorophenyl)but-3-en-2-one (2d)**

***
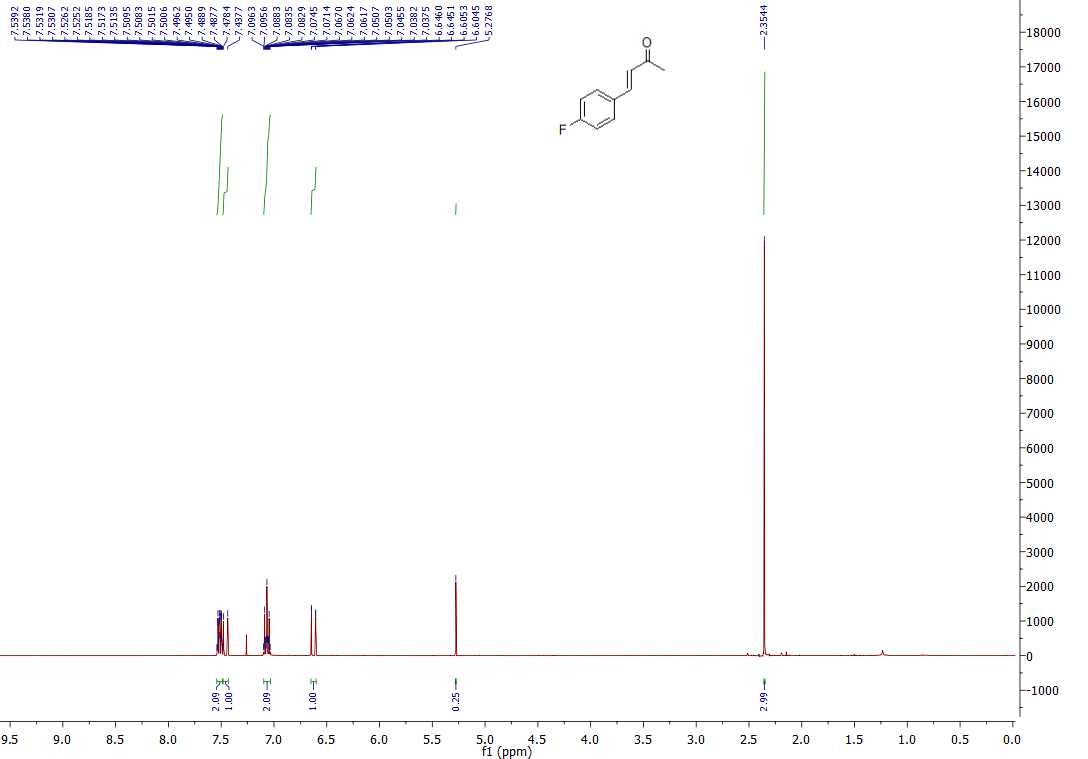
***

***(E)-*4-(4-bromophenyl)but-3-en-2-one (2e)**

***
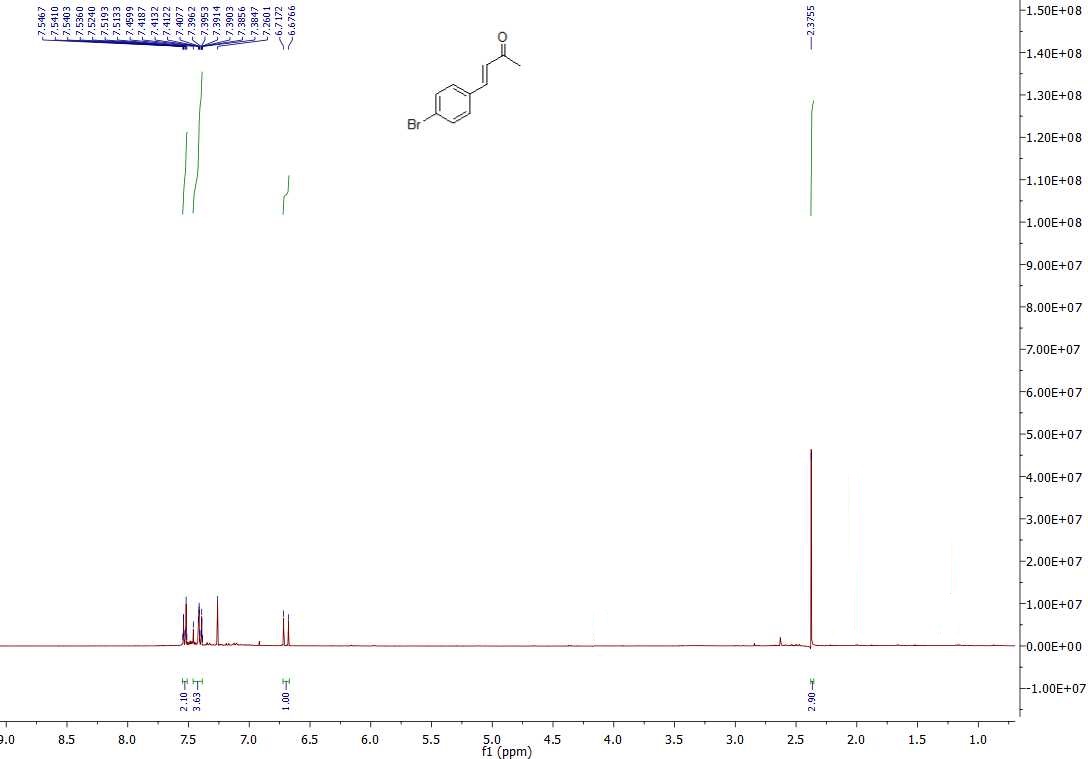
***

***(E)-*4-(4-methoxyphenyl)but-3-en-2-one (2f)**

***
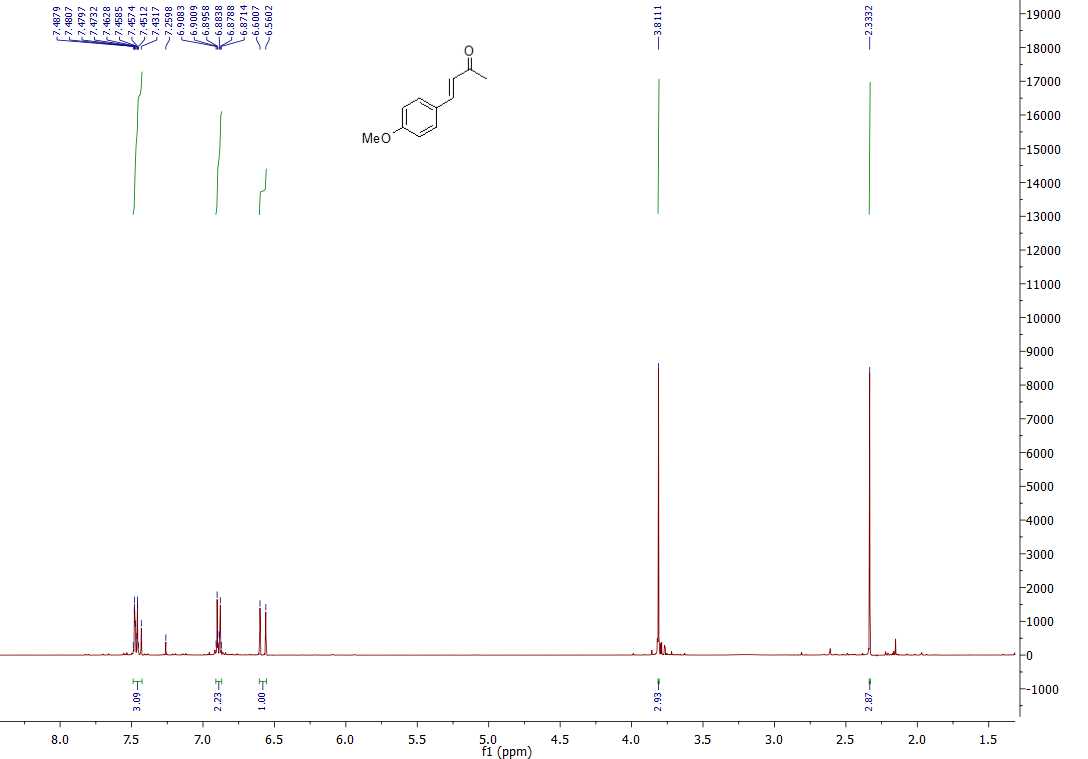
***

***(E)-*4-(3-chlorophenyl)but-3-en-2-one (2g)**

***
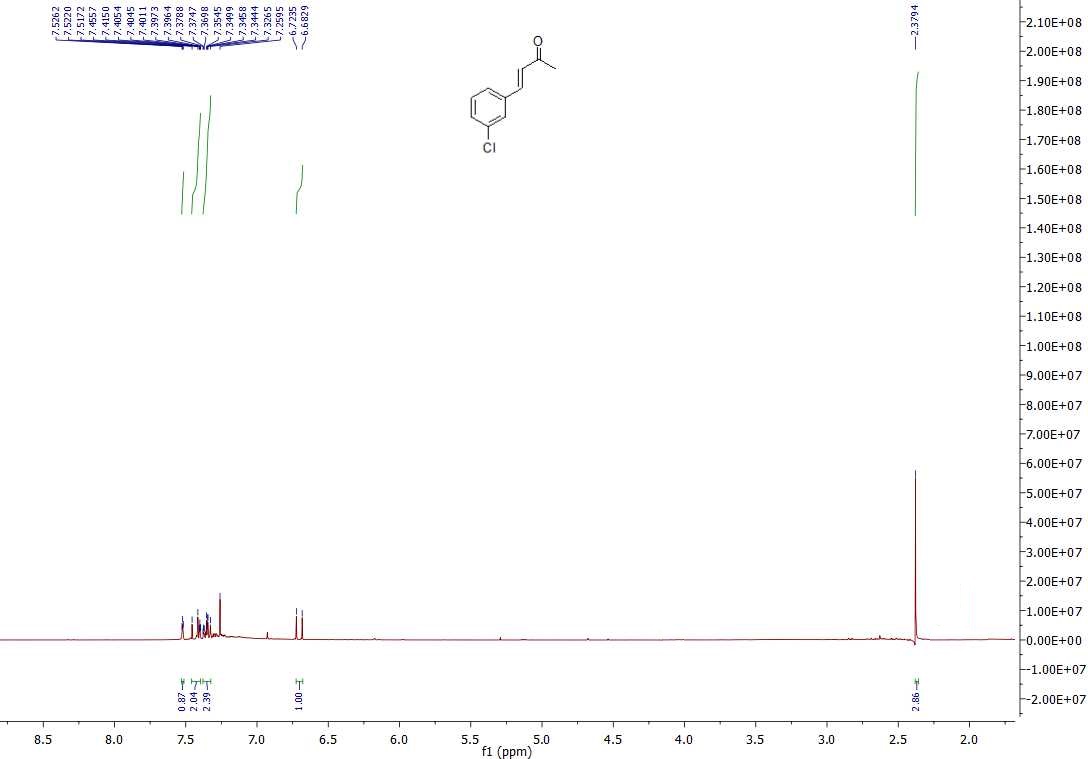
***

***(E)-*4-(4-nitrophenyl)but-3-en-2-one (2h)**

***
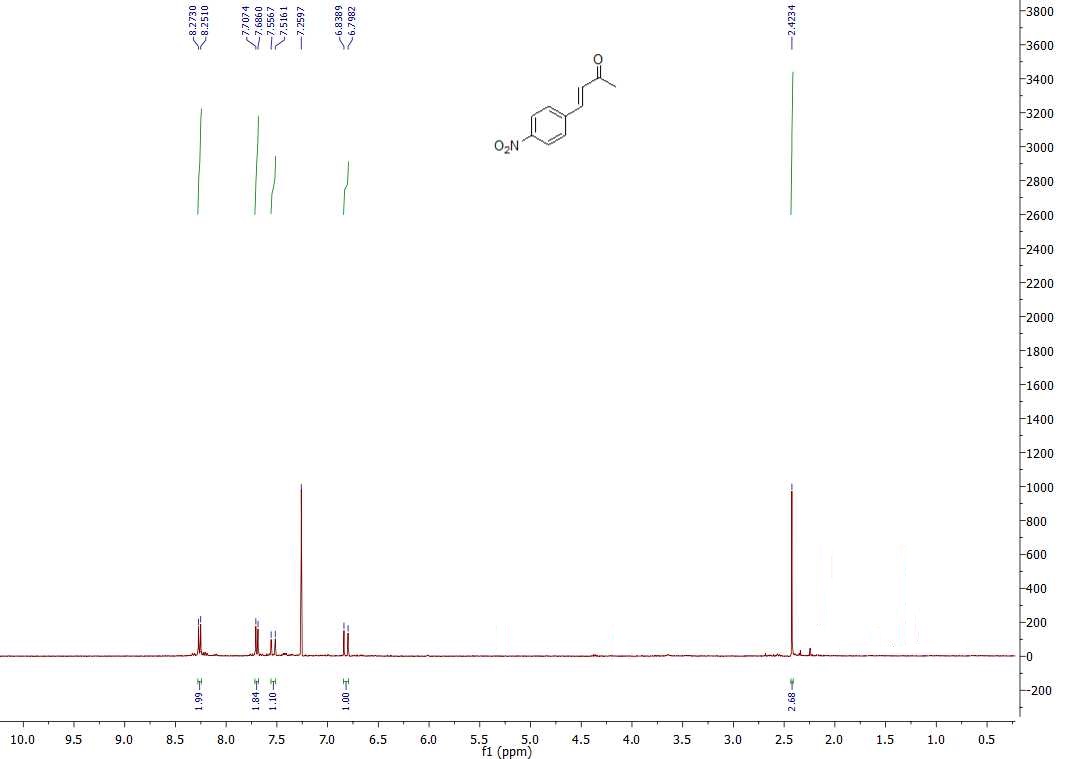
***

***(E)-*4-(4-(trifluoromethyl)phenyl)but-3-en-2-one (2i)**

***
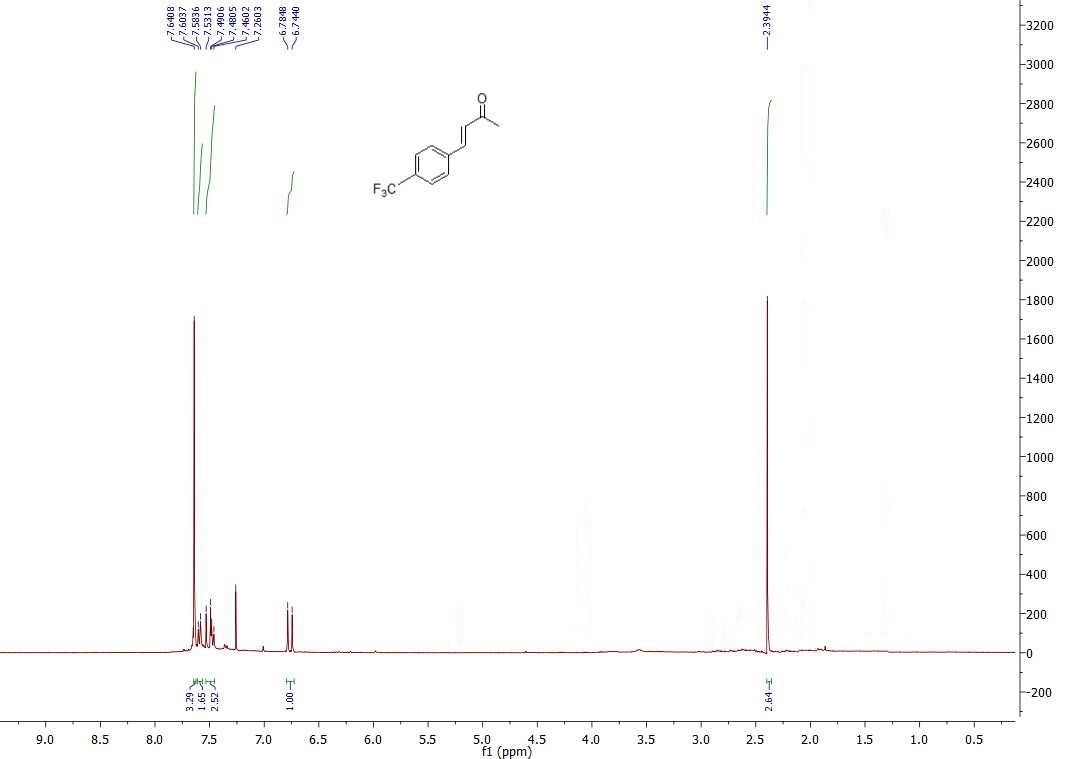
***

***(E)-*4-(3,4-dimethoxyphenyl)but-3-en-2-one (2j)**

***
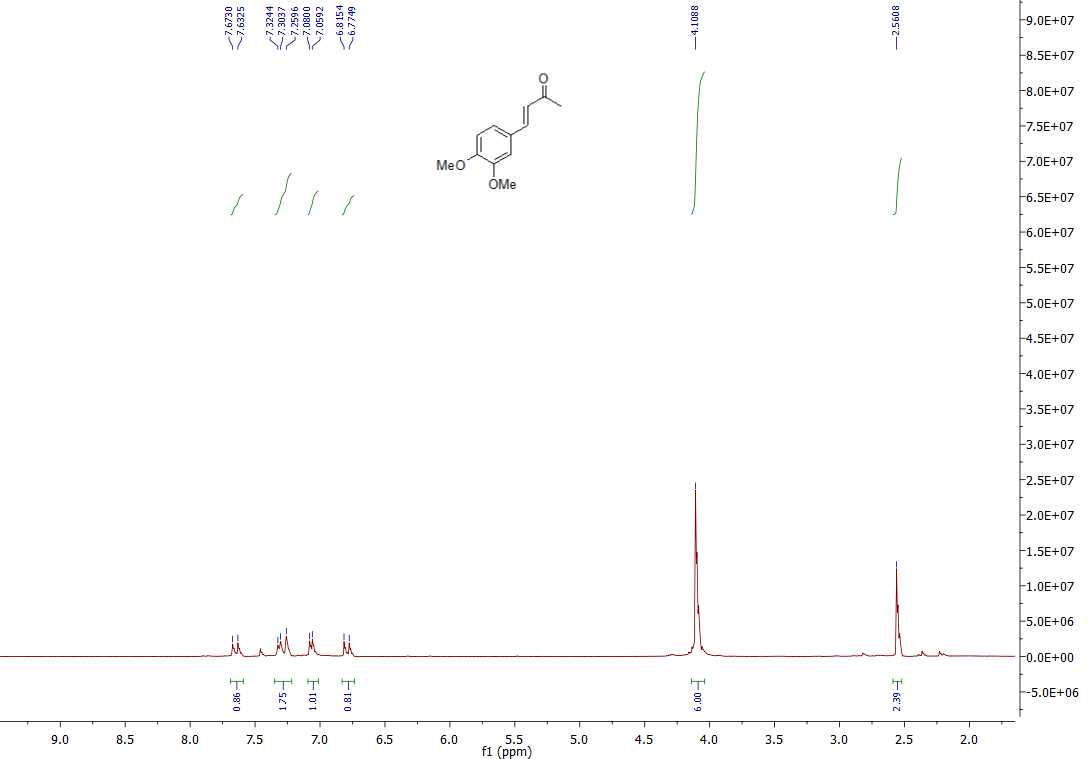
***

***(E)-*4-(5-chloro-2-nitrophenyl)but-3-en-2-one (2k)***

**
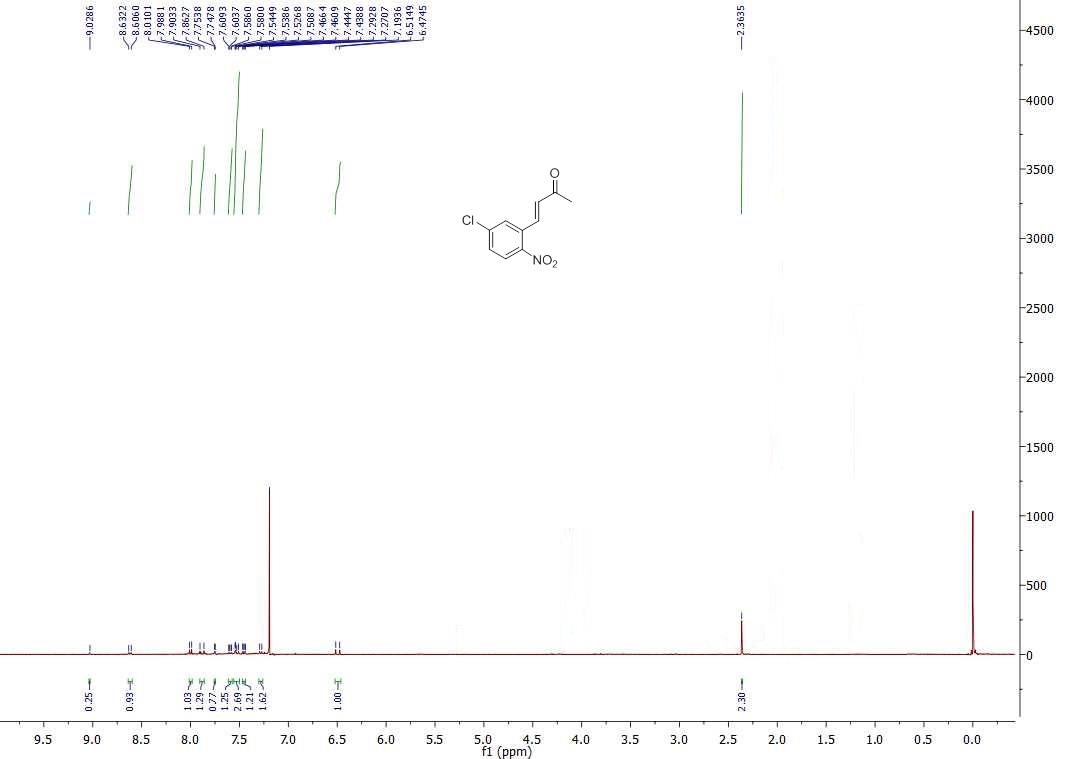
**

*****12% of non-reacted aldehyde was recovered (Table 2, entry 5)

***(E)-*4-(naphthalen-1-yl)but-3-en-2-one (2l)**


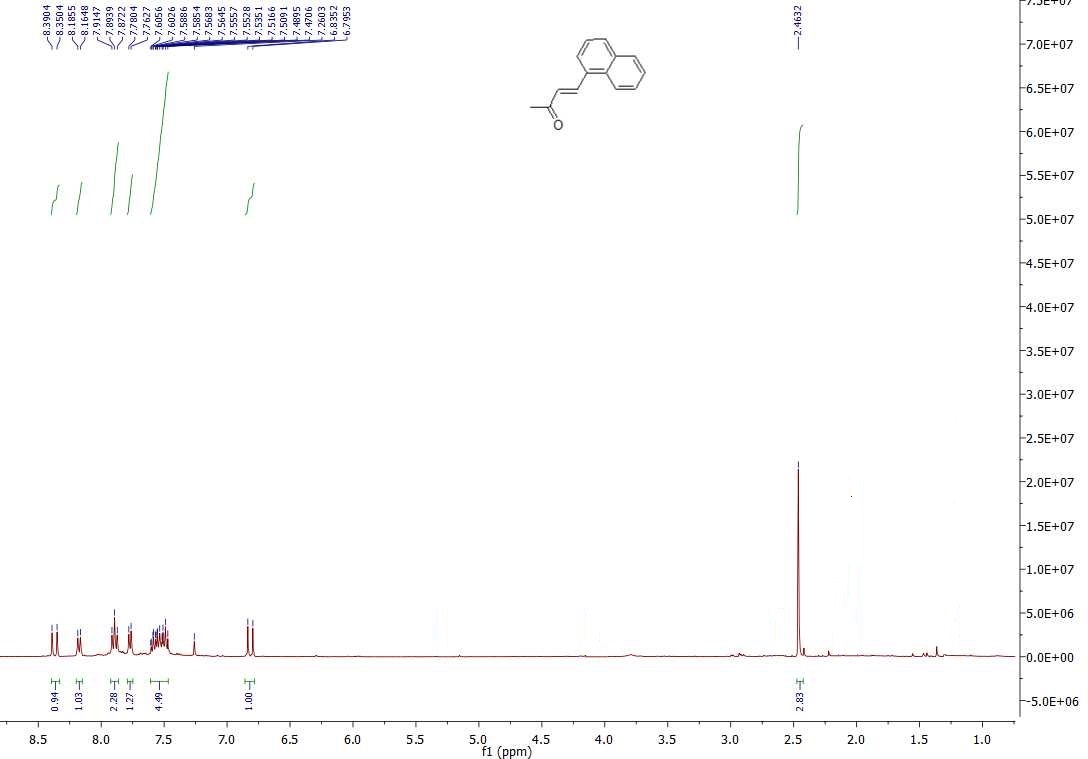

Supplement: Additional file 5: — Proton NMR spectra of compounds 2a-2l. [file 40064_2015_985_MOESM5_ESM.docx]
